# Interplay between 4-Hydroxy-3-Methyl-2-Alkylquinoline and *N*-Acyl-Homoserine Lactone Signaling in a *Burkholderia cepacia* Complex Clinical Strain

**DOI:** 10.3389/fmicb.2017.01021

**Published:** 2017-06-20

**Authors:** Annelise Chapalain, Marie-Christine Groleau, Servane Le Guillouzer, Aurélie Miomandre, Ludovic Vial, Sylvain Milot, Eric Déziel

**Affiliations:** ^1^CIRI, Centre International de Recherche en Infectiologie, Equipe Pathogénèse des Légionelles, Inserm, U1111, Université Claude Bernard Lyon 1, CNRS, UMR5308, École Normale Supérieure de Lyon, Université LyonLyon, France; ^2^INRS-Institut Armand-Frappier, LavalQC, Canada; ^3^CNRS, INRA, UMR 5557, Ecologie Microbienne, Université Lyon 1Villeurbanne, France

**Keywords:** quorum sensing, *Burkholderia ambifaria*, gene regulation

## Abstract

Species from the *Burkholderia cepacia* complex (Bcc) share a canonical LuxI/LuxR quorum sensing (QS) regulation system named CepI/CepR, which mainly relies on the acyl-homoserine lactone (AHL), octanoyl-homoserine lactone (C_8_-HSL) as signaling molecule. *Burkholderia ambifaria* is one of the least virulent Bcc species, more often isolated from rhizospheres where it exerts a plant growth-promoting activity. However, clinical strains of *B. ambifaria* display distinct features, such as phase variation and higher virulence properties. Notably, we previously reported that under laboratory conditions, only clinical strains of the *B. ambifaria* species produced 4-hydroxy-3-methyl-2-alkylquinolines (HMAQs) *via* expression of the *hmqABCDEFG* operon. HMAQs are the methylated counterparts of the 4-hydroxy-2-alkylquinolines (HAQs) produced by the opportunistic human pathogen *Pseudomonas aeruginosa*, in which they globally contribute to the bacterial virulence and survival. We have found that unlike *P. aeruginosa*’s HAQs, HMAQs do not induce their own production. However, they indirectly regulate the expression of the *hmqABCDEFG* operon. In *B. ambifaria*, a strong link between CepI/CepR-based QS and HMAQs is proposed, as we have previously reported an increased production of C_8_-HSL in HMAQ-negative mutants. Here, we report the identification of all AHLs produced by the clinical *B. ambifaria* strain HSJ1, namely C_6_-HSL, C_8_-HSL, C_10_-HSL, 3OHC_8_-HSL, 3OHC_10_-HSL, and 3OHC_12_-HSL. Production of significant levels of hydroxylated AHLs prompted the identification of a second complete LuxI/LuxR-type QS system relying on 3OHC_10_-HSL and 3OHC_12_-HSL, that we have named CepI2/CepR2. The connection between these two QS systems and the *hmqABCDEFG* operon, responsible for HMAQs biosynthesis, was investigated. The CepI/CepR system strongly induced the operon, while the second system appears moderately involved. On the other hand, a HMAQ-negative mutant overproduces AHLs from both QS systems. Even if HMAQs are not classical QS signals, their effect on AHL-based QS system still gives them a part to play in the QS circuitry in *B. ambifaria* and thus, on regulation of various phenotypes.

## Introduction

Cell–cell communication in bacteria occurs *via* the production of signal molecules that are released and captured in the micro-environment. When the bacterial population grows, the local concentration of signals increases, until it reaches a threshold able to trigger regulatory cascades. This communication based on the census of the population is named quorum sensing (QS) and it allows the coordination of collective behaviors such as the production of virulence factors or biofilm formation (Fuqua and Winans, 1994; Williams, 2007). As one of the first described QS-regulated phenotype was the production of luminescence by *Aliivibrio fischeri*, the synthase and the regulator were named LuxI and LuxR, respectively. Similar systems described thereafter have been consequently named LuxI/LuxR-type proteins (Fuqua and Winans, 1994; Whitehead et al., 2001).

In Gram-negative bacteria, QS often relies on signal molecules belonging to the acyl-homoserine lactone (AHL) family (Williams, 2007), which are produced by LuxI-type synthases. The general model for QS regulation is that at a threshold concentration, the AHL binds a cognate LuxR-type regulator, leading to the induction or repression of target genes, including the AHL synthase itself, thus creating an auto-inducing loop (Suarez-Moreno et al., 2012b). A bacterium can possess one or more QS system, that could act independently, hierarchically or in opposition, in order to achieve finely tuned regulation. This has been extensively studied in *Pseudomonas aeruginosa*, a human opportunistic pathogen, which possesses numerous virulence factors that are mostly controlled by QS (Jimenez et al., 2012). The QS circuitry of this bacterium is composed of two LuxI/LuxR-type systems, named LasI/LasR and RhlI/RhlR. Besides these two QS systems based on signal molecules belonging to the AHL family, *P. aeruginosa* possesses a third QS system relying on 4-hydroxy-2-alkylquinolines (HAQs) molecules. The three QS systems in *P. aeruginosa* are hierarchically organized but intertwined; for example, the two AHL-based systems directly or indirectly induce the *pqsABCDE* operon, required for HAQ production, while some HAQs autoinduce their own biosynthesis but do not influence the production of AHLs (Déziel et al., 2005).

Bacterial species belonging to the *Burkholderia cepacia* complex (Bcc) carry a canonical LuxIR-system named CepIR that mainly relies on C_8_-HSL as signaling molecule (Lutter et al., 2001; Venturi et al., 2004). Additional LuxIR-type systems have been described in some Bcc species, such as BviIR in *Burkholderia vietnamiensis*, or CciIR in epidemic strains of *Burkholderia cenocepacia* (Malott et al., 2005; Malott and Sokol, 2007). As a member of the Bcc, *Burkholderia ambifaria* expresses a CepIR system, and associated phenotypes have been identified, such as production of proteases and antifungal and antimicrobial compounds (Zhou et al., 2003; Chapalain et al., 2013).

4-Hydroxy-2-alkylquinolines were thought to be exclusively produced by *P. aeruginosa* until they were also detected in cultures of a few *Burkholderia* species (Diggle et al., 2006). We later determined that at least three *Burkholderia* species, namely *B. pseudomallei* and *B. thailandensis*, which belong to the pathogenic *Burkholderia pseudomallei*-*thailandensis*-*mallei* group, and *B. ambifaria*, a member of the pathogenic Bcc group, mostly produce HAQs harboring an unsaturated alkyl side chain and a methyl group at the 3′ position, thus referred to as 4-hydroxy-3-methyl-2-alkylquinolines (HMAQs) (Vial et al., 2008). These differences are due to the last two genes of the *hmqABCDEFG* operon in *Burkholderia*, while the first five genes of the operon are homologous to *pqsABCDE* (Vial et al., 2008; Dulcey et al., 2013). Indeed *hmqF* is responsible for the unsaturation of the alkyl chain whereas *hmqG* codes for a probable methyltransferase required for the methylation of HMAQs (Vial et al., 2008; Agarwal et al., 2012).

The only function ascribed so far to HMAQs is as antifungal compounds (Kilani-Feki et al., 2011). However, besides acting as QS autoinducers, several roles have been attributed to HAQs from *P. aeruginosa*, including immunomodulatory properties but these have not been tested for HMAQs yet (Hooi et al., 2004; Skindersoe et al., 2009). We have previously reported that only clinical strains of *B. ambifaria* are able to produce HMAQs, and that this production negatively impacts the biosynthesis of C_8_-HSL (Vial et al., 2008, 2010). In the present study, we wanted to better understand the links between QS and regulation of HMAQs in *B. ambifaria*. To do so, we have used the clinical strain HSJ1, isolated from sputum of a cystic fibrosis patient, to explore the AHL-based QS circuitry and its intertwinement with the HMAQ system.

## Materials and Methods

### Bacterial Strains and Culture Conditions

The bacterial strains used in this study are listed in **Table 1**. Unless otherwise stated, all strains were routinely grown at 37°C in Tryptic Soy broth (TSB) (BD), with shaking (240 rpm) in a TC-7 roller drum (New Brunswick). *B. ambifaria* HSJ1 cultures were inoculated from freshly grown colonies on TSB plates solidified with 1.5% agar and containing 0.1% Congo Red to avoid picking phase variants (Vial et al., 2010). Tetracycline was used at 15 and 200 μg/mL for *Escherichia coli* and *B. ambifaria* HSJ1, respectively, while trimethoprim was included at 100 μg/mL for both species. TSB was supplemented with 62.5 μg/mL diaminopimelic acid (DAP) for growth of auxotrophic *E. coli* χ7213.

**Table 1:** Bacterial strains used in this study.

Strains	Description	Reference
***B. ambifaria***		
ED336	HSJ1, wild-type strain, isolated from a cystic fibrosis patient	Vial et al., 2008
ED372	*cepI*::pKnock-Cm mutant in HSJ1, Cm^R^	Chapalain et al., 2013
ED358	*cepR*::pKnock-Cm mutant in HSJ1, Cm^R^	Chapalain et al., 2013
ED2132	*cepI2*::*tp* mutant in HSJ1, Tp^R^	This study
ED350	*hmqA*::pKnock-Cm mutant in HSJ1, Cm^R^	Vial et al., 2008
ED2114	HSJ1*::hmqA-lacZ*, Tet^S^	This study
ED2117	HSJ1 *hmqA*-::*hmqA-lacZ*, Cm^R^, Tet^S^	This study
ED2118	HSJ1 *cepI*-::*hmqA-lacZ*, Cm^R^, Tet^S^	This study
ED2116	HSJ1 *cepI2*-::*hmqA-lacZ*, Tp^R^, Tet^S^	This study
ED2115	HSJ1 *cepR*-::*hmqA-lacZ*, Cm^R^, Tet^S^	This study
ED2133	*cepR2*::*tp* mutant in HSJ1, Tp^R^	This study
ED2134	*cepI* ::pKnock-Cm, *cepI2 ::tp* mutant in HSJ1, Cm^R^, Tp^R^	This study
ED2136	HSJ1 *cepI-cepI2*-::*hmqA-lacZ*, Cm^R^, Tp^S^, Tet^S^	This study
ED2138	HSJ1 *cepR2*-::*hmqA*-*lacZ*, Tp^S^, Tet^S^	This study
***E. coli***		
DH5α	Φ80 *lacZ*ΔM15 (*lacZYA*-*argF*) *U169 hsdR17* (*r_k_-*, *m_k_+*) *recA1 endA1 supE44 thi-1 gyrA relA1*	Invitrogen
SM10 (λ*pir*)	*thi thr leu tonA lacY supE recA*::RP4–2-Tc::Mu Km λ*pi*r	Simon et al., 1983
χ7213	thi-1 thr-1 leuB6 glnV44 fhuA21 lacY1 recA1 RP4-2-Tc::Mu λ*pir* ΔasdA4 Δzhf-2::Tn1	Kang et al., 2002

*Cm, chloramphenicol; Tet, tetracycline; Tp, trimethoprim*.

### Plasmids

The plasmids used in this study are listed in **Table 2**. All primers used for the constructions were purchased from Alpha DNA (Montreal, QC, Canada) and are listed in Supplementary Table 1.

**Table 2:** Plasmids used in this study.

Plasmids	Description	Reference
Mini-CTX-*lacZ*	Integration vector for insertion of promoter-*lacZ* fusions in the CTX attachment site, Tet^R^	Becher and Schweizer, 2000
pJPD01	Region upstream *hmqA* inserted in EcoRI-BamHI site in Mini-CTX-*lacZ*, Tet^R^	This study
pEX18Tet-*pheS*	Gene replacement vector, Tet^R^	Barrett et al., 2008
pFTP1	source of Tp^R^ FRT cassette, Ap^R^, Tp^R^	Choi et al., 2005
pAC1	*cepI2*::Tp inserted in HindIII site in pEX18Tet-*pheS*	This study
pMCG27	*cepR2*::Tp inserted in HindIII site in pEX18Tet-*pheS*	This study
pFLPe4	FRT site-specific excision vector, contains *rhaS*-*rhaR*-*P_rhaBAD_*-*FLPe*, Ap^R^ Km^R^	Choi et al., 2008

*Tet, tetracycline; Tp, trimethoprim; Km, kanamycin; Ap, ampicillin*.

### Construction of the *cepI2*-, *cepR2*-, and *cepI*-*cepI2*- Mutants

The *cepI2*- marked mutant of *B. ambifaria* strain HSJ1 was constructed essentially using the method described by Barrett et al. (2008). Briefly, one upstream and one downstream fragments of the Bamb_6053 locus (AMMD strain sequence, assembly GCF_000203915.1, from www.burkholderia.com) were amplified by PCR using the Bamb6053_02F/Bamb6053_02R and Bamb6053_03F/Bamb6053_03R2 primer pairs, respectively (Supplementary Table 1). A FRT-flanked trimethoprim resistance cassette was amplified by PCR from the pFTP1 donor plasmid (Choi et al., 2005) using primers Bamb6053_Trim01F2 and Bamb6053_Trim01R2. The three PCR products were joined together using the Bamb6053_02F/Bamb6053_03R2 primer pair and Taq DNA polymerase (Feldan). The resulting fragment was digested using FastDigest HindIII (ThermoScientific) and cloned into pEX18Tet-*pheS* (Barrett et al., 2008). The resulting pAC1 construction was introduced into *E. coli* SM10 (λ*pir*) allowing conjugation with *B. ambifaria* HSJ1. Transformants were selected onto agar plates supplemented with tetracycline and trimethoprim. Merodiploids were resolved by successive subcultures in TSB with trimethoprim only. Tetracycline-sensitive/trimethoprim-resistant colonies were selected on TSB agar plates supplemented with appropriate antibiotics and then PCR-confirmed. The pAC1 construct was introduced in the HSJ1 *cepI*- background (ED372) to generate the *cepI*-*cepI2*- marked double mutant.

The *cepR2*- mutant was constructed using the same methodology. Primers Bamb6040_02F and 02R and Bamb6040_03F and _03R were used to respectively amplify regions upstream and downstream of the Bamb_6040 locus in strain HSJ1. The trimethoprim resistance cassette from pFTP1 was amplified with primers Bamb6040_01F2 and 01R. The three fragments were joined together by amplification using Bamb6040_02F and Bamb6040_03R. The resulting fragment was purified and digested with HindIII and ligated in pEX18Tet-*PheS* digested with the same enzyme, to generate the pMCG27 construct. Double crossing-over in *B. ambifaria* HSJ1 was performed using subcultures on M9 agar plates with 0.1% *p*-chlorophenylalanine, 0.2% dextrose and 100 μg/mL trimethoprim, then verified as described above.

### Construction of Chromosomally Integrated *hmqA*-*lacZ* Reporter in *B. ambifaria* HSJ1

Amplification of the intergenic region upstream of the *hmqA* gene was carried out using primers *hmqA*A-L and *hmqA*A-R containing respectively EcoRI and BamHI restriction sites. The P*hmqA* PCR product was ligated with T4 DNA ligase (BioBasic, Inc.) in EcoRI/BamHI-digested mini-CTX-*lacZ* (Becher and Schweizer, 2000) to generate pJPD01. Chromosomal integration of the mini-CTX-*hmqA*-*lacZ* reporter at the *attB* locus in *B. ambifaria* strain HSJ1, and in *hmqA*-, *cepI*-, *cepR*-, *cepI2*-, *cepR2*-, and *cepI-cepI2*- mutants was performed by mating with donor strain *E. coli* χ7213 harboring pJPD01. An overnight culture of each mutant (recipient) was diluted in fresh broth and incubated with agitation at 37°C until an OD_600_ of 0.5 was reached. An overnight culture of donor *E. coli* χ7213 (pJPD01) was also diluted in fresh DAP-containing TSB and statically grown at 37°C to an OD_600_ of 0.5. Volumes of 1.5 mL from each culture were centrifuged at 8,000 ×*g* and both pellets were resuspended together in 100 μL PBS. The whole volume was then spotted onto an LB agar plate containing 100 μg/mL DAP and incubated overnight at 30°C. The bacteria were then suspended in 1 mL PBS and spread on TSB agar plates containing tetracycline. Tetracycline-resistant conjugants were selected and insertion of the *hmqA*-*lacZ* fusion was confirmed by PCR. Finally, an unmarked insertion was obtained by Flp-mediated excision of the tetracycline cassette, using pFLPe4 (Choi et al., 2005).

### HMAQ Purification

To obtain a quantity of 4-hydroxy-3-methyl-2-heptenylquinoline (HMAQ-C_7_:2′), the main HMAQ produced by *B. ambifaria* (Vial et al., 2008), 6 L of *B. ambifaria* HSJ1 were grown until stationary phase in TSB. Three liters of methanol were then added to the culture and cells were removed by centrifugation at 6,000 ×*g* for 25 min. After methanol evaporation using a Rotovapor R110 (Büchi, Switzerland), the supernatant was extracted three times with 1.5 L ethyl acetate. The extracts were pooled, dehydrated with Na_2_SO_4_ and filtered on 6 μm glass fiber. Complete evaporation was obtained using the Rotovapor and the residue was solubilized in 10 mL methanol. Purification was performed by HPLC (Waters Delta Prep 4000) on a Gemini C18 column (10 μm, 110 Å, 50^∗^21.2 mm, Phenomenex) with a linear gradient of acetonitrile/water (neutralized to pH 7.0 with NH_4_OH). The chromatographic fractions containing HMAQ-C_7_:2′ were pooled, evaporated and suspended in 1 mL isopropanol. Twenty mL pentane was added for overnight precipitation at 16°C. The mixture was filtered on 6 μm glass fiber filter and air dried completely. LC/MS was used to assess purity during the process.

### LC/MS–MS Analyses for AHL and HMAQ Production

The samples were prepared and analyzed as previously described (Chapalain et al., 2013), with the following differences: the positive electrospray ionization (ESI+) mode, supplemented by the multiple reactions monitoring (MRM) mode were used and the following transitions were monitored: HHQ-d4: 148→163; C_6_-HSL: 200→102; C_8_-HSL: 228→102; 3OH-C_8_-HSL: 244→102; C_10_-HSL: 256→102; HMAQ-C_7_:2′: 256→172; 3OH-C_10_-HSL: 272→102; HMAQ-C_9_:2′: 284→172; 3OH-C_12_-HSL: 300→102. AHL concentrations were calculated from integration of peak areas, expressed in relative value compared to the internal standard 5,6,7,8-tetradeutero-4-hydroxy-2-heptylquinoline (HHQ-d4) as before (Lepine and Deziel, 2011).

### Quantification of β-Galactosidase Activity in *B. ambifaria* HSJ1

Levels of expression from the *hmqABCDEFG* promoter were assessed using strains carrying the chromosomal *lacZ* transcriptional fusion. β-Galactosidase assays were performed as described (Miller, 1972). For experiments with addition of AHLs, C_8_-HSL (Sigma-Aldrich), 3-OH-C_8_-HSL and 3-OH-C_10_-HSL (Nottingham) stocks were prepared in HPLC-grade acetonitrile. *B. ambifaria* HSJ1::*hmqA*-*lacZ* cultures were grown in TSB from freshly picked colonies. Cultures were then diluted to an OD_600_ = 0.1 in TSB and 10 μM AHL of interest were added. Acetonitrile only was added in controls. For experiments with addition of HMAQs, cultures were prepared as above and HMAQs were added to cultures to a final concentration of 50 μM from stocks prepared in HPLC-grade methanol. Methanol was added in controls. β-Galactosidase activity was measured at various time intervals during growth. All experiments were performed with four replicates and repeated at least twice.

### Quantitative Reverse-Transcription Polymerase Chain Reaction (qRT-PCR) Experiments

Samples were prepared as previously described (Chapalain et al., 2013). Primers used for *cepI*, *cepI2*, and *hmqA* mRNA quantification are shown in Supplementary Table 1, using the method described before (Chapalain et al., 2013). The reference gene was *ndh* (Subsin et al., 2007). Gene expression differences between HSJ1 WT and *cepR* and *cepR2* or *hmqA*- mutants were calculated using the 2^-ΔΔCT^ formula (Livak and Schmittgen, 2001).

### Data Analysis

Unless otherwise stated, data are reported as mean ± standard deviation (SD). Statistical analyses were performed with the R software v.3.3.3^1^ using one-way analysis of variance (ANOVA). Probability values less than 0.05 were considered significant.

## Results

### HMAQs Do Not Directly Induce Their Own Production

In *P. aeruginosa*, the HAQs HHQ and PQS are able to activate the transcription of the *pqsABCDE* operon *via* their binding to the MvfR (PqsR) regulator (Xiao et al., 2006). In *B. ambifaria* HSJ1, no such regulator has been identified (Vial et al., 2008). However, we have reported that an *hmqG*- mutant, which is only able to produce non-methylated HAQs, produces lower concentrations of total HAQs compared to the WT strain (only 25–30% of WT), thus raising the possibility of a positive regulation of HMAQs on their own production, likely via *hmqABCDEFG* regulation (Vial et al., 2008).

**FIGURE 1:**
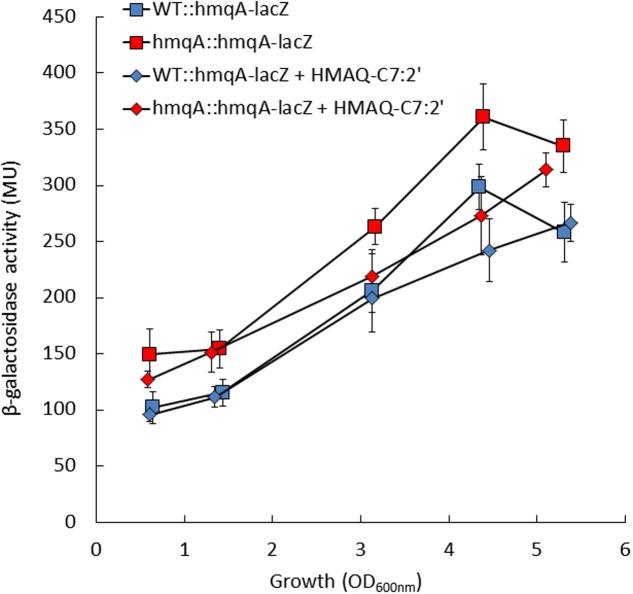
Transcription levels from the *hmqABCDEFG* promoter in *Burkholderia ambifaria* HSJ1 wild-type and *hmqA-* mutant. β-Galactosidase activities of an *hmqA-lacZ* chromosomal reporter were monitored during the growth of the WT and *hmqA-* mutant strains, in presence or absence of added 50 μM HMAQ-C_7_:2′. Results are expressed in Miller units (MU) as means ± SD of four replicates.

To verify this hypothesis, the expression of the *hmqABCDEFG* operon was monitored using the *hmqA*-*lacZ* reporter gene in WT and *hmqA-* mutant strains cultures, supplemented or not with HMAQ-C_7_:2′, the principal HMAQ produced by *B. ambifaria* HSJ1. The β-galactosidase activity in the *hmqA*- mutant was not abolished, and was even significantly higher than in the WT strain on all time points (*p* < 0.05) (**Figure 1**). Supplementation with HMAQ-C_7_:2′ had no effect on transcription from the *hmqA* promoter in the WT strain, whereas it significantly (*p* < 0.05) restored activity to the WT level in *hmqA*- for the late time points (**Figure 1**). The same results were obtained with addition of HMAQ-C_9_:2′, the second most abundant congener produced in our strain (data not shown). Collectively these results indicate that, unlike in *P. aeruginosa*, HMAQs do not activate transcription of the operon responsible for their synthesis in *B. ambifaria* HSJ1, and at best are poor negative signals on the *hmq* system.

### A Mutant Deficient for HMAQ Biosynthesis Overproduces CepI-Derived AHLs and Additional 3-Hydroxylated AHLs

**FIGURE 2:**
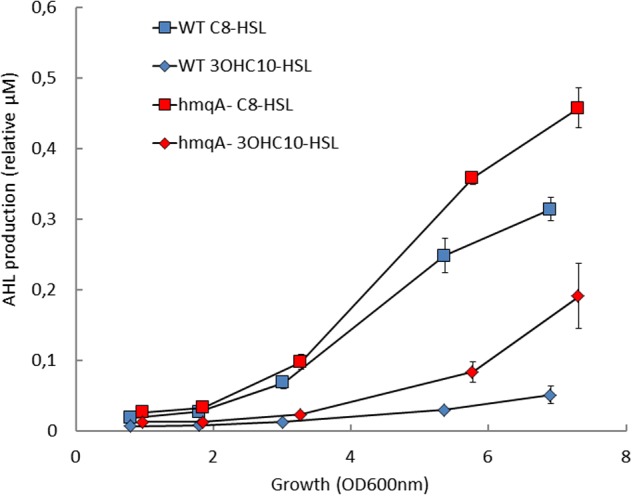
Acyl-homoserine lactone (AHL) production in the *hmqA-* mutant of *B. ambifaria* HSJ1. WT and *hmqA-* were analyzed for C_8_-HSL and 3OHC_10_-HSL production in growing cultures using LC/MS. Values are presented relative to the internal standard HHQ-d4. Data are expressed as means ± SD of four replicates.

In a precedent study, we had demonstrated that the *hmqA*- mutant of *B. ambifaria* HSJ1 overproduces C_8_-HSL, the main QS molecule that was then known in Bcc species (Vial et al., 2008). Although C_8_-HSL is the most abundant AHL produced by Bcc bacteria, other AHLs can also be produced by *Burkholderia*. Based on the reported AHLs produced by *Burkholderia* species (Suarez-Moreno et al., 2010; Majerczyk et al., 2013) and since it was previously reported, however using only TLC assays, that *B. ambifaria* produces more diversified AHLs than simply C_6_-HSL and C_8_-HSL (Lutter et al., 2001), the following AHLs were investigated: C_4_-HSL, C_6_-HSL, C_8_-HSL, C_10_-HSL, 3OHC_8_-HSL, 3OHC_10_-HSL, 3OHC_12_-HSL, 3oxoC_8_-HSL, 3oxoC_12_-HSL, and 3oxoC_14_-HSL. Under our conditions, HSJ1 indeed mostly produces C_8_-HSL (**Table 3**). However, unexpectedly and interestingly, the next more abundant AHLs are 3-hydroxylated, especially 3OHC_10_-HSL. We saw neither C_4_-HSL, nor any oxo-substituted AHLs. In agreement with our previous data, levels of all these new AHLs were also significantly higher in cultures of the *hmqA*- when compared to the WT (*p* < 0.05) (**Figure 2** and data not shown).

**Table 3:** Acyl-homoserine lactone (AHL) production (nM) in LuxI-type synthases mutants of *B. ambifaria* HSJ1.

Strains	C_4_-HSL	C_6_-HSL	C_8_-HSL	3OH-C_8_-HSL	C_10_-HSL	3OHC_10_-HSL	3OC_12_-HSL	3OHC_12_-HSL
HSJ1	*ND*	8.5 ± 1.13	281.3 ± 58.3	11.35 ± 1.67	4.96 ± 1.39	50.39 ± 6.85	*ND*	1.32 ± 0.80
*cepI*-	*ND*	*ND*	*ND*	0.128 ± 0.09	*ND*	10.19 ± 1.75	*ND*	*ND*
*cepI2*-	*ND*	9.22 ± 2.87	275.75 ± 13.94	10.28 ± 3.23	5.02 ± 1.04	0.69 ± 0.20	*ND*	*ND*

*The six AHLs identified in the WT strain have been quantified using LC/MS in supernatants of the *cepI-* and *cepI2-* mutant strains at the stationary phase (OD_600_= 5–6). ND, not detected; Data are expressed as means ± SD of three replicates*.

When production of these AHLs in the *cepI*- mutant was investigated we confirmed that while C_6_-HSL, C_8_-HSL, and C_10_-HSL production was clearly abolished and thus directly depend on this synthase (Chapalain et al., 2013), 3OHC_10_-HSL and small concentrations of 3OHC_8_-HSL were still detected (**Table 3**). The same results were obtained with the *cepR*- mutant (data not shown).

### Identification of *cepI2*, a New AHL Synthase-Coding *luxI* Homolog

The above results pointed to the presence of at least a second AHL synthase in our strain. We have previously used the *B. ambifaria* strain AMMD sequenced genome to identify genes in strain HSJ1 (Winsor et al., 2008; Chapalain et al., 2013). In AMMD, predictions indicate that the Bamb_6053 locus on the third chromosome encodes a putative AHL synthase, with homology to *bviI* from *B. vietnamiensis* G4 (54.92% identity) and to *btaI2* from *B. thailandensis* E264 (65.54% identity). A multiple sequence alignment of Bamb_6053 with various AHL synthases shows that the predicted synthase possesses eight residues that are conserved within the LuxI AHL synthase family and are needed for AHL synthesis (Watson et al., 2002) (Supplementary Figure 1).

We knocked-out the Bamb_6053 gene in our HSJ1 strain, which we named *cepI2*, resulting in the almost complete loss of 3OHC_10_-HSL production, while 3OHC_8_-HSL remained unaffected (**Table 3**). On the other hand, the *cepI2*- mutant still showed C_6_-HSL, C_8_-HSL, and C_10_-HSL levels similar to the WT strain (**Table 3**). Furthermore, a *cepI*-*cepI2*- mutant does not produce any known AHLs, confirming that there is no other synthase in this strain (data not shown). We conclude that the clinical *B. ambifaria* HSJ1 strain expresses two AHL synthases: the canonical CepI responsible for C_8_-HSL, C_6_-HSL, C_10_-HSL and 3OH-C_8_-HSL, and CepI2 which is mainly responsible for 3OH-C_10_-HSL and 3OH-C_12_-HSL synthesis.

### Identification of CepR2, the Cognate LuxR Regulator of *cepI2*

Different types of organization of the QS *luxI/R* family genes are reported, including in *Burkholderia* (Choudhary et al., 2013). In strain AMMD, no cognate *luxR*-type gene was identified associated with *cepI2* (Choudhary et al., 2013). To identify such cognate regulator for *cepI2*, the genetic proximity between *luxI* and *luxR* genes often is a clue. However, the closest *luxR*-type genes are located either 13 genes upstream (Bamb_6040) or 11 genes downstream (Bamb_6064) from the *cepI2* locus. Using the InterPro database, we quickly discarded Bamb_6064 as a potential candidate, since it possesses only the DNA binding domain of a LuxR-type regulator (IPR000792 entry) and not the autoinducer binding domain (IPR005143 entry), characteristic of a LuxR-type regulator that interacts with AHLs (Subramoni et al., 2015). Moreover, nine residues are conserved in the LuxR family (Egland and Greenberg, 2001; Koch et al., 2005); six of them involved in ligand binding. The remaining three are involved in DNA binding. Bamb_6040 possesses the nine residues conserved in the LuxR family (Supplementary Figure 2), while in Bamb_6064 some key residues are replaced. A phylogenetic analysis of described LuxR from the *Burkholderia* genus, including Bamb_6040 and LuxR references from other species is presented in Supplementary Figure 3. This analysis shows that Bamb_6040 is clustered with and closer to BviR from *B. vietnamiensis* G4 and *B. cepacia* DB01 (with whom it shares 41% of identity) than to BtaR2 from *B. thailandensis*. However, these three are more closely related than to CepR or BtaR1 (Supplementary Figure 3).

**FIGURE 3:**
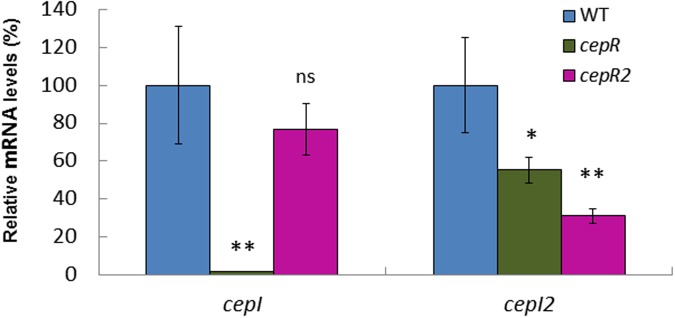
Expression of the two synthases in *B. ambifaria* HSJ1. The relative mRNA levels for *cepI* and *cepI2* genes were measured at the end of log phase (OD_600_ = 4–5) in WT, *cepR*- mutant and *cepR2*- mutant. Data are expressed as means of at least three values ± SD. Analysis of variance (ANOVA) were performed using R and Tukey fit comparisons. ^∗∗^*p* < 0.01; ^∗^*p* < 0.05; ns, non-significant.

In order to confirm that Bamb_6040 encodes the cognate LuxR of *cepI2*, a mutant was constructed in our strain HSJ1 and studied for AHL production. The mutant still produced similar concentrations of C_8_-HSL compared to the WT, but drastically reduced concentrations of 3OHC_10_-HSL (Supplementary Figures 4A,B). Complementation with a plasmid-borne *cepR2* restored 3OHC_10_-HSL production to WT levels (data not shown). Our qRT-PCR experiments confirmed that expression of the *cepI2* gene was lowered in the Bamb_6040 mutant compared to the WT, compatible with the level of decrease of 3OHC_10_-HSL (**Figure 3**). Since these elements support Bamb_6040 as the cognate LuxR-type regulator of *cepI2* we named it CepR2.

### Phenotypic Characterization of *cepI2*/*cepR2* Mutants

Several phenotypes previously reported to be affected in the *cepI*- mutant (Chapalain et al., 2013) have been evaluated in the *cepI2*- and *cepR2*- mutants, such as protease production, hemolytic and antifungal activities. Other known phenotypes were also included in the characterization, such as resistance to antibiotics, biofilms formation, and virulence against macrophages or amoeba (Vial et al., 2010). We observed that the *cepR2*- mutant displayed a higher proteolytic activity than the WT (Supplementary Figure 5A), while it was significantly reduced in *cepI*- and in the AHL-defective *cepI*-*cepI2*- mutant. For cytotoxicity against macrophages (Supplementary Figure 5B), *cepR2*- mutant displayed a moderate but significant decrease in cytotoxicity compared to the WT against a human monocytes/macrophages cell line, whereas *cepI*- and *cepI*-*cepI2*- were both importantly affected. For the other phenotypes tested, *cepI2*- and *cepR2*- mutants displayed no significant differences with the WT. In all our assays, *cepR*- displayed the same pattern as *cepI*- (data not shown).

In order to more globally evaluate the involvement of the second QS system in strain HSJ1, we undertook competition assays. We used the *cepR*- and *cepR2*- mutants to avoid chemical complementation with AHLs produced by the WT strain. The competition conducted in human monocytes/macrophages THP-1 cell line revealed that the *cepR*- mutant displayed the same ability as the WT to enter in macrophages (2 h post-infection), while it was less competitive in replication (evaluation at 8 h post-infection) (Supplementary Figure 5C). The *cepR2*- mutant displayed a slight decrease in competitive index to enter in macrophages, and was also less competitive to replicate (Supplementary Figure 5C, left panel). The defect was more pronounced in the *cepR*- mutant than in the *cepR2*- mutant, nevertheless both systems seemed to be required in this environment. As for the competition in pea rhizosphere, only the *cepR*- mutant was less recovered from roots compared to the WT, providing the evidence that only the first QS system is essential in this environment (Supplementary Figure 5, right panel).

### AHL-Mediated Quorum Sensing Positively Regulates HMAQ Biosynthesis

**FIGURE 4:**
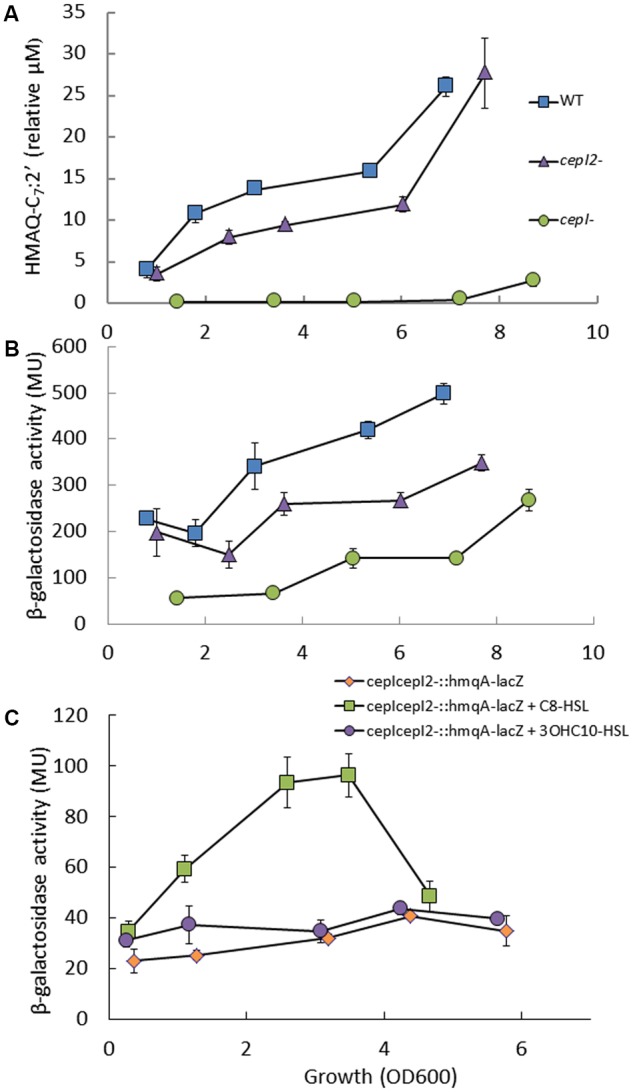
4-Hydroxy-3-methyl-2-alkylquinolines (HMAQs) production and *hmqABCDEFG* operon expression in wild-type and mutants strains of *B. ambifaria* HSJ1. **(A)** HMAQ-C_7_:2′ production in WT*, cepI- and cepI2-* mutant strains using LC/MS. Results are presented as relative concentrations compared to the internal standard HHQ-d4 and expressed as means ± SD of four replicates. **(B**) β-Galactosidase activities of an *hmqA-lacZ* chromosomal reporter were monitored during the growth of the WT, *cepI- and cepI2-* mutant strains. Results are expressed in Miller units (MU) as means ± SD of three replicates. **(C)** Activation of *hmqABCDEFG* expression by AHLs. β-Galactosidase activities were monitored in *cepI-cepI2-*::*hmqA-lacZ* cultures, supplemented with 10 μM C_8_-HSL or 10 μM 3OH-C_10_-HSL. Results are expressed as means ± SD of three replicates.

As the *hmqA*- mutant strain overproduces AHLs synthetized by both CepI and CepI2, we wondered whether the production of HMAQs was reciprocally affected in QS mutants of *B. ambifaria*, as seen for HAQs in *P. aeruginosa* (Déziel et al., 2004). Strain HSJ1 produces a mix of HMAQs, the most abundant being HMAQ-C_7_:2′, while the polar *hmqA*- mutant produces no HMAQ (Vial et al., 2008). We thus determined the kinetic of production of HMAQ-C_7_:2′ in the different mutants described above vs. the WT strain. The *cepI*- (**Figure 4A**) and *cepR*- (data not shown) mutants no longer produce HMAQs. On the other hand, the *cepI2*- mutant displays only a moderate, although statistically significant decrease in HMAQ-C_7_:2′ production (*p* < 0.05) (**Figure 4A**). AHL-mediated QS affect directly and indirectly the transcription of the *pqsABCDE* operon in *P. aeruginosa* (McGrath et al., 2004; Xiao et al., 2006). An *hmqA*-*lacZ* reporter allowed us to follow the transcription from the *hmqABCDEFG* operon promoter in the different strains described above (**Figure 4B**). Expression of *hmqA*-*lacZ* is reduced in *cepI2-* mutant background, and is even lower in the *cepI*- mutant (**Figure 4B**), which is consistent with the HMAQ measurements (**Figure 4A**). Taken together, these results suggest an influence of the AHL-based regulon on HMAQ production.

To further discriminate the role of both AHL QS systems in the transcription of *hmqABCDEFG*, we then assessed the activity of our *hmqA-lacZ* reporter in the AHL-defective *cepI*-*cepI2*- double mutant upon supplementation with one of the two main AHL produced by each synthase, namely C_8_-HSL and 3OHC_10_-HSL. We measured no effect of 3OHC_10_-HSL on the activity of *hmqA*-*lacZ*, whereas C_8_-HSL strongly induced expression above the level of the control (*p* < 0.05) (**Figure 4C**). Accordingly, supplementation of the *cepI*- mutant with C_8_-HSL restored HMAQ production (Supplementary Figure 6A). This was also confirmed at the transcriptional level as the *hmqA* gene is almost 50 times downregulated in the *cepI*- mutant strain compared to the WT (Supplementary Figure 6B). The expression was partially restored if the mutant culture is supplemented with C_8_-HSL. Although these results collectively indicate that the CepIR QS system is essential for the expression of the *hmqABCDEFG* operon, and that C_8_-HSL is the most potent AHL for this induction, we have been unable to show a direct interaction between CepR and the *hmqABCDEFG* promoter when co-expressed together in a heterologous host system (data not shown).

## Discussion

### AHL Circuitry in *B. ambifaria* HSJ1

Like the other Bcc species, *B. ambifaria* possesses a canonical CepI synthase responsible for C_8_-HSL and C_6_-HSL production (Zhou et al., 2003; Venturi et al., 2004; Chapalain et al., 2013). Other AHLs produced by *B. ambifaria*, then known as genomovar VII, were detected previously using TLC plates, but they could not be identified (Lutter et al., 2001). Performing an exhaustive search using LC/MS–MS analyses, we have found that strain HSJ1 produces additional AHLs, namely C_10_-HSL, 3OH-C_8_-HSL, 3OH-C_10_-HSL, and 3OHC_12_-HSL. We have also identified a second AHL synthase gene that we have named *cepI2* primarily responsible for production of the latter two.

The two AHL synthases produce sets of mostly non-overlapping AHLs (**Table 3**). Although 3OHC_8_-HSL seems produced by both LuxI homologues, CepI is clearly the main synthase for this AHL. At first glance it was surprising that CepI produces a mixture of carbonyl- and hydroxyl-AHL. If a synthase could theoretically produce a variety of AHLs, they often differ by the length of their acyl chain but not by the third carbon-borne substitution (Watson et al., 2002). Nevertheless, there are examples in the literature reporting synthases able to produce a mix of AHLs harboring different substitutions (Wisniewski-Dye et al., 2002; Niu et al., 2008). The mechanism by which a synthase recognizes and discriminates its favorite acyl-ACP is far to be fully understood, even if recent study reports progress in this respect for *B. mallei* BmaI1 (Montebello et al., 2014). For example the 140th amino acid of the synthase sequence was reported to determine the substitution borne by the third carbon, as a threonine appeared required to produce 3-oxo-AHL (Watson et al., 2002). Neither CepI nor CepI2 has a threonine at this position in their sequence; accordingly, we did not find any oxo-AHL in supernatants of strain HSJ1.

As the 3OHC_10_-HSL levels were similar between the *cepI* and *cepR* mutant strains (data not shown), we suspected that there was a LuxR-type transcriptional regulator other than CepR involved with *cepI2* regulation. A previous study on the QS genes organization in *Burkholderia* has reported that the AMMD strain possesses a potential synthase, but no cognate LuxR-type homolog was identified (Choudhary et al., 2013). Indeed, while no obvious LuxR-type transcriptional regulator is found in the close vicinity of the Bamb_6053 locus, our *in silico* analysis predicted Bamb_6040 to be the best candidate for *cepI*2 regulation. The inactivation of this gene, renamed *cepR2*, confirmed this hypothesis.

**FIGURE 5:**
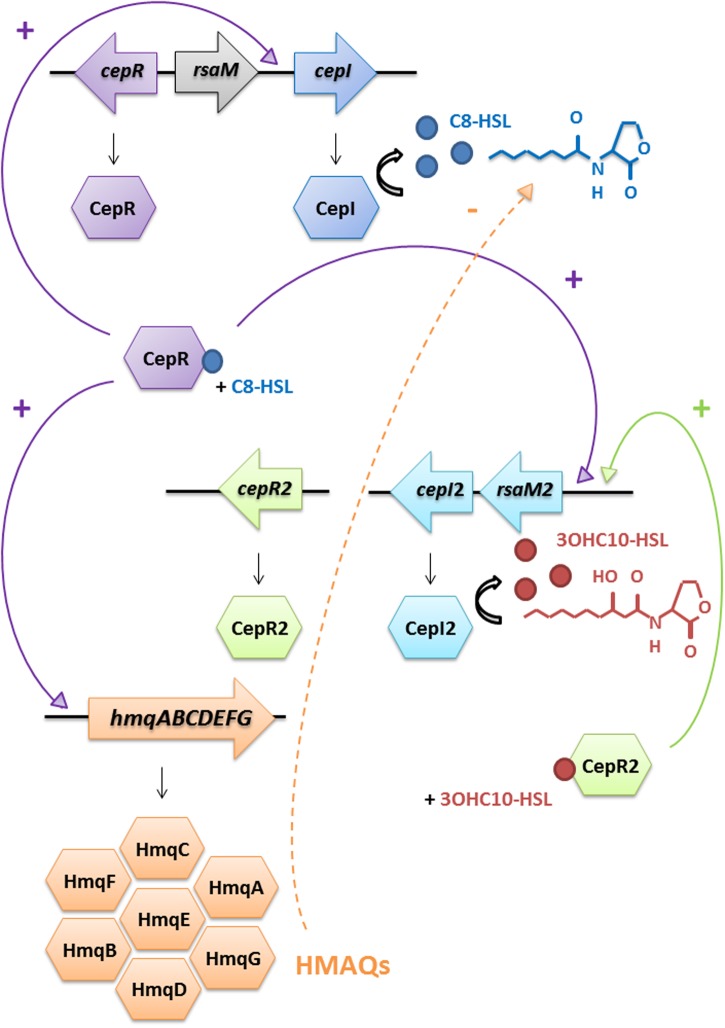
Schematic representation of the interactions between AHL-based QS systems and HMAQ production in *B. ambifaria*. The CepI/CepR system directly activates the *cepI* gene. The CepI/CepR system also activates the *hmqABCDEFG* operon expression and thus HMAQ biosynthesis. HMAQs inhibit AHLs production from both synthases, probably by acting on the CepI/CepR system, exploiting the hierarchical organization of CepIR on CepI2R2. HMAQs do not induce their own production. Confirmed regulation is indicated by a solid arrow and proposed regulation is indicated by a dashed arrow.

As 3OH-C_10_-HSL production in the *cepI* mutant is lowered compared to the WT (**Table 3**), we propose a hierarchical influence of CepR on *cepI2* (**Figure 5**), CepI being the producer of the main AHLs recognized by CepR (Weingart et al., 2005). The kinetics of C_8_-HSL and 3OH-C_10_-HSL productions in the WT strain displayed in **Figure 2** supports this hypothesis, showing that C_8_-HSL accumulation starts earlier and faster than 3OH-C_10_-HSL. Such hierarchical relationships between two or more AHL-based QS systems have already been described in other Gram-negative bacteria, such as LasIR and RhlIR systems in *P. aeruginosa*, or CciIR in *B. cenocepacia* (O’Grady et al., 2009; Jimenez et al., 2012). We identified a similar organization in *B. thailandensis* E264 (Le Guillouzer et al., unpublished). The observation that the CepIR system is well-conserved in Bcc species suggests its early acquisition (Suarez-Moreno et al., 2012a); we could thus expect CepIR to be the primary system modulating additional AHL-based QS modules. However, examples in other Bcc suggest that the second QS system can be integrated in a species or even strain-dependent manner, rendering any hierarchical relationship difficult to predict (Malott and Sokol, 2007; O’Grady et al., 2009).

Accordingly, it was difficult to identify phenotypes clearly attributed only to the CepI2R2 system. The competition assay in macrophages revealed conditions where the second QS system seems to be required. Interestingly, the CepIR system appeared to be essential only in the rhizosphere. Consistently with this result, we did not observe antifungal activities depending on the second QS system, while the first system is clearly essential (data not shown) (Zhou et al., 2003; Chapalain et al., 2013). It is possible that the implication of the CepI2R2 system is condition-dependent. The determination of genes and phenotypes controlled either by CepIR or CepI2R2, cooperatively, independently or in opposition will require a global and comprehensive approach, such as those undertaken for *B. cenocepacia* or *B. thailandensis* (O’Grady et al., 2009; Majerczyk et al., 2014a).

### An Homeostatic Interplay between AHL and HMAQ Signaling

To study the reciprocal effect of AHL on the *hmqABCDEFG* operon transcription in HSJ1, we performed experiments using diverse tools and mutants, including *hmqA*-*lacZ* reporter assays correlated to LC/MS measurements of AHL and HMAQ production.

We verified whether HMAQs can act as classical signaling molecules implicated in an autoinducing loop in *B. ambifaria* HSJ1, similarly to the situation in *P. aeruginosa*. Indeed, this bacterium also possesses two AHL-based QS systems intertwined with a HAQ-based QS system. The *pqsABCDE* operon is directly and indirectly influenced by the two AHL-based QS systems, but is also positively autoinduced *via* the binding of HHQ or PQS ligands to the LysR-type regulator MvfR (PqsR) (McGrath et al., 2004; Xiao et al., 2006). While no such regulator has yet been identified for *B. ambifaria* (Vial et al., 2008), we investigated whether HMAQ-C_7_:2′ induces the transcription of the *hmqABCDEFG* operon in strain HSJ1. The transcriptional activity from the *hmq* promoter did not decrease in the *hmqA*- mutant. Actually, especially considering the negative effect of the exogenous addition of HMAQ-C_7_:2′ at certain time points in the cultures, it even seems that HMAQs and/or the *hmq* system slightly downregulates its expression, likely indirectly. It is noteworthy that we observe the same scenario in an *hmqA*- mutant from HMAQ-producer strain *B. thailandensis* E264 (Le Guillouzer et al., unpublished). This result was unexpected because in complete contrast with the situation seen with the *pqs* system in *P. aeruginosa*, where a *pqsA-* mutant strain exhibits a very low *pqsABCDE* operon transcriptional activity, which is induced by addition of HHQ, or even better PQS (Xiao et al., 2006). It is possible that other regulatory components present in the operon are responsible for the observed effect on the transcription in the *hmqA*- mutant. In *P. aeruginosa*, the *pqsABCDE* operon is upregulated in a *pqsE*- mutant (Hazan et al., 2010). It is possible HmqE might have a similar impact in *B. ambifaria* since both proteins seem to be functionally complementary (Diggle et al., 2006).

We knew that the *hmqA-* mutant of *B. ambifaria* HSJ1 overproduces C_8_-HSL, the main AHL then known, already revealing a link between QS and HMAQ production (Vial et al., 2008). Now that the QS circuitry is better understood, we checked whether production of other AHLs is affected by the absence of *hmqA* and, indeed, we found that all AHLs from both synthases are overproduced in the *hmqA-* mutant. Accordingly, we observed an upregulated expression of *cepI2* in the *hmqA*- mutant using qRT-PCR (Supplementary Figure 7). However, expression of *cepI* was similar to the WT in both exponential and stationary growth phases. This was surprising considering the observed effect on C_8_-HSL levels.

One explanation could be that HMAQs act as inhibitors of CepI activity or C_8_-HSL function, which would then affect *cepI2* transcription via diminished CepR activity (**Figure 5**). Further supporting this model, the addition of HMAQ-C_7_:2′ in the AHL-defective double *cepI*-*cepI2*- mutant had no effect on *hmqA*-*lacZ* activity (data not shown), while it downregulated transcription from the *hmqA* promoter in a HMAQ-negative background, where CepI is overproducing C_8_-HSL (**Figure 1**). These results confirm that even if the *hmqABCDEFG* operon impacts its own expression, HMAQs do not have autoinducing properties in *Burkholderia*. Since the *hmqA*- mutant is polar on downstream genes (Vial et al., 2008), we do not know if what we observed is due to the absence of HMAQ molecules or of another gene of the *hmq* operon. In *P. aeruginosa* HAQs do not impact AHL production (Déziel et al., 2005), highlighting another difference between systems.

Recent transcriptomic data suggest that globally QS downregulates the expression of the *hmqABCDEFG* operon in two other HMAQ-producing *Burkholderia* species, *B. pseudomallei* and *B. thailandensis* (Majerczyk et al., 2014a,b). In *B. ambifaria*, the CepIR QS system acts as a positive regulator of the *hmqABCDEFG* operon (**Figure 5**), highlighting an intriguing difference in HMAQ regulation between Bcc and *pseudomallei*-*thailandensis* groups. In the *cepI-cepI2*- background of *B. ambifaria* HSJ1, the main product of CepI C_8_-HSL very efficiently induces transcription from the *hmq* promoter. Still, despite the clear implication of the CepIR system in the induction of the operon, a direct regulation of CepR on the *hmqABCDEFG* promoter was not observed using an heterologous host strategy, presumably because QS impact on HMAQ production implicates additional regulatory elements. Investigating HMAQ production and *hmqABCDEFG* transcription revealed that the *cepI2-* mutant displays a statistically significant lower transcriptional activity than the WT strain, with a well-correlated decreased HMAQ production (**Figures 4A,B**). However, 3OHC_10_-HSL produced *via* CepI2 did not increase the transcriptional activity above the level of the control in the *cepI-cepI2*- mutant. Together with the observed upregulation of *cepI2* expression in the *hmqA*- mutant, these results suggest that the CepI2R2 system would affect indirectly the transcription of *hmqABCDEFG via* an effect on the CepIR system.

Some other observations lead to suggest that QS is not the only regulon controlling the production of HMAQs in *B. ambifaria*. First, although phase variants of HSJ1 express a functional CepIR system and produce similar amount of C_8_-HSL compared to the WT, no HMAQ production is observed (Vial et al., 2010). Secondly, under our experimental conditions, environmental strains which possess the operon, such as the AMMD strain, do not produce HMAQs (Vial et al., 2010). However, another team has demonstrated that AMMD can produce some HMAQs, using experimental conditions that also favor antifungal and antimicrobial molecules production (Mahenthiralingam et al., 2011). This collectively suggests the presence of additional regulatory levels on HMAQ production dependent on the environmental conditions, maybe at a post-transcriptional level or *via* another regulator yet to be discovered. Supplemental experiments are thus needed to completely decipher the regulation of HMAQ biosynthesis. Since the *hmqABCDEFG* operon seems to be present in many now sequenced Bcc strains (burkholderia.com), there is still a lot to be learned on the functionality and regulation of this operon. Even if we have contributed to better understand HMAQ regulation, their role in *B. ambifaria* HSJ1 is still largely unknown. It has been proposed that HMAQs are QS-controlled secondary metabolites (Mahenthiralingam et al., 2011; Majerczyk et al., 2014b). Indeed, in another Bcc species, namely *B. cepacia*, HMAQs have been described as antifungals (Kilani-Feki et al., 2011). This property cannot be easily verified in HSJ1 as the *hmqA*- mutant overproduces all the AHLs, resulting in an overexpression of all the QS-regulated phenotypes including antifungal compounds, masking the antifungal contribution of HMAQs.

## Conclusion

In this study we have contributed to better understand the AHL circuitry in *B. ambifaria*, and its intertwinement with HMAQs. We have identified a second LuxIR-type QS system in the HSJ1 strain, and we have characterized AHLs produced by each synthase. We have also revealed that QS, essentially *via* the CepIR system and C_8_-HSL, induces HMAQ production, while the second system is moderately involved. In contrast, a mutant defective for HMAQ production accumulates AHLs produced by both synthases. These elements plead in favor of a non-traditional inducing loop summarized in **Figure 5**. However, even if HMAQs are not classical QS molecules, their negative impact on AHL-based QS suggests that they are probably more than secondary metabolites in *B. ambifaria* HSJ1. Since HMAQs seem to be specifically produced by *B. ambifaria* clinical strains, at least under the conditions tested (Vial et al., 2010), it is possible that their involvement in an infection context is more important than we know. Investigations on the production of HMAQs in other clinical Bcc strains could lead to a better understanding of their role as a potential virulence determinants.

## Author Contributions

All authors conceived and designed the experiments. AC, M-CG, SLG, AM, and SM performed the experiments. All authors analyzed the data. All authors contributed to writing, editing and finalizing the manuscript.

## Conflict of Interest Statement

The authors declare that the research was conducted in the absence of any commercial or financial relationships that could be construed as a potential conflict of interest.

## Footnotes

1 http://www.R-project.org
