# Supplementary material for: Interplay between 4-Hydroxy-3-Methyl-2-Alkylquinoline and N-Acyl-Homoserine Lactone Signaling in a Burkholderia cepacia Complex Clinical Strain
Source: Front Microbiol. 2017 Jun 20;8:1021. doi: 10.3389/fmicb.2017.01021 (PMC5476693; doi:10.3389/fmicb.2017.01021)
Supplement: Supplementary file 1 [file Data_Sheet_1.pdf]

Supplementary Material

Material and Methods

Phenotypic assays

Phenotypic assays were performed as previously described (Sokol et al., 1979;Vial et al., 2010;Chapalain et al., 2013). Protease activity was evaluated on BHI-milk agar (Sokol et al., 1979). Competition assays against human monocytes/macrophages THP-1 cell line were conducted as previously described (Vial et al., 2010) except that a MOI = 5 was used instead of 10. Competition assays in pea rhizosphere (*Pisum sativum*) were conducted as previously described (Vial et al., 2010) with the following modifications: roots were sampled 8 days after inoculation and bacteria were collected by 2 min vortexing. Cytotoxicity against human monocytes/macrophages THP-1 cells line was estimated using cells differentiated for 48h with Phorbol 12-myristate 13-acetate (PMA, Sigma). The supernatants were collected after 24h infection at a MOI = 1 and cytotoxicity was determined by measuring the lactate dehydrogenase (LDH) activity using the CytoTox 96® Non-Radioactive Cytotoxicity Assay kit (Promega). Values were calculated as % compared to a positive lysis control.

Phylogenetic analysis

The sequences of LuxR proteins with predicted autoinducer binding domain and LuxI proteins of *B. ambifaria* strain AMMD, were obtained from the InterPro database (entry IPR005143 and IPR001690 respectively). Sequences of highly similar *luxR* and *luxI* genes were obtained by using blast tool (Kent, 2002) and aligned against TraR (*A. tumefaciens* C58) and EsaI (*Pantoea stewartii*) with Muscle (Edgar, 2004). Detection of all key residues previously reported to be invariant in several functional LuxR proteins have been sought with respect to TraR of *Agrobacterium tumefaciens* (W57, Y61, D70, P71, W85, G113, E178, L182, G188) and LuxI proteins with respect to EsaI of *P. stewartii* (Arg24, Phe28, Trp34, Asp45, Asp48, Arg68, Glu97, Arg100) (Egland and Greenberg, 2001;Watson et al., 2002;Koch et al., 2005). Phylogenetic analyses were performed by using the Maximum Likelihood method based on the LG matrix-based model (Le and Gascuel, 2008), which generated trees with the highest log likelihood. Significance was estimated by using Bootstrap analysis.

Supplementary Table 1: Primers used in this study

Primers	Description	Reference
**hmqAA-L**	AAGAATTCATCCCGACTAGCTGGTGATG	This study
**hmqAA-R**	CGCGGATCCGTTCTTCGACTGCGGTATCC	This study
**ndhF**	GCGATCGGGCTGTACAAGTT	Subsin, 2007
**ndhR**	AGTGGCTCAGCGACTGGAA	Subsin, 2007
**hmqARTF**	GCCGCTACCTGAATGACACG	This study
**hmqARTR**	ACCGTGTAGCCGGAGCTGAT	This study
**qBafIF**	TTTCTCGCGAACACGTAGACGGTA	This study
**qBafIR**	GTGTATTCGTCGAACAGCTCGGTTG	This study
**SLG_qRTcepI2_F**	GACCGGACACGATCCATATC	This study
**SLG_qRTcepI2_R**	CCGAGGATGTAGGACGAAAA	This study
**Bamb6053_02F**	AAGCTTCGAAATCCAGCCATTCATGGC	This study
**Bamb6053_02R**	GTGCATGTGCGAACTCCAATG	This study
**Bamb6053_03F**	AGCTGAAATTGGGCCGGCTG	This study
**Bamb6053_03R2**	AAGCTTCGGTACGCGATGTAGTTCAG	This study
**Bamb6053_Trim01F2**	TCCTCCATTTCGCATTTAATGCGTACGTCCATTGGAGTTC GCACATGCACCCGAGCTCGAATTGGGGATCTT	This study
**Bamb6053_Trim01R2**	CGTCGCGCGGGACGGCATGCGTTGGGACCGCAGCCGGC CCAATTTCAGCTCGAGCTCGAATTAGCTTCAAA	This study
**Bamb6040_02F_**	AAGCTTGTCGACAACGCTCGACTACA	This study
**Bamb6040_02R**	TTTCGAATACATCGTCGAAGC	This study
**Bamb6040_03F**	ATTTCGGATCTCGAACTTGC	This study
**Bamb6040_03R**	AAGCTTACGTGCGTAATGGACTGTGA	This study
**Bamb6040_01F2**	CGTCATGACGACGTTGTCGCGCGCGCTGAGCTTCGACGA TGTATTCGAAACCGAGCTCGAATTGGGGATCTT	This study
**Bamb6040_01R2**	GCCAGATAGATCAGCCGGCTTTCCGTGGCGGCAAGTTCG AGATCCGAAATCGAGCTCGAATTAGCTTCAAA	This study

References

Chapalain, A., Vial, L., Laprade, N., Dekimpe, V., Perreault, J., and Deziel, E. (2013). Identification of quorum sensing-controlled genes in *Burkholderia ambifaria*. *Microbiologyopen* 2**,** 226-242.

Edgar, R.C. (2004). MUSCLE: multiple sequence alignment with high accuracy and high throughput. *Nucleic Acids Res* 32**,** 1792-1797.

Egland, K.A., and Greenberg, E.P. (2001). Quorum sensing in *Vibrio fischeri*: analysis of the LuxR DNA binding region by alanine-scanning mutagenesis. *J Bacteriol* 183**,** 382-386.

Kent, W.J. (2002). BLAT--the BLAST-like alignment tool. *Genome Res* 12**,** 656-664.

Koch, B., Liljefors, T., Persson, T., Nielsen, J., Kjelleberg, S., and Givskov, M. (2005). The LuxR receptor: the sites of interaction with quorum-sensing signals and inhibitors. *Microbiology* 151**,** 3589-3602.

Le, S.Q., and Gascuel, O. (2008). An improved general amino acid replacement matrix. *Mol Biol Evol* 25**,** 1307-1320.

Sokol, P.A., Ohman, D.E., and Iglewski, B.H. (1979). A more sensitive plate assay for detection of protease production by *Pseudomonas aeruginosa*. *J Clin Microbiol* 9**,** 538-540.

Subsin, B., Chambers, C.E., Visser, M.B., and Sokol, P.A. (2007). Identification of genes regulated by the cepIR quorum-sensing system in *Burkholderia cenocepacia* by high-throughput screening of a random promoter library. *J Bacteriol* 189**,** 968-979.

Vial, L., Groleau, M.C., Lamarche, M.G., Filion, G., Castonguay-Vanier, J., Dekimpe, V., Daigle, F., Charette, S.J., and Deziel, E. (2010). Phase variation has a role in *Burkholderia ambifaria* niche adaptation. *ISME J* 4**,** 49-60.

Watson, W.T., Minogue, T.D., Val, D.L., Von Bodman, S.B., and Churchill, M.E. (2002). Structural basis and specificity of acyl-homoserine lactone signal production in bacterial quorum sensing. *Mol Cell* 9**,** 685-694.

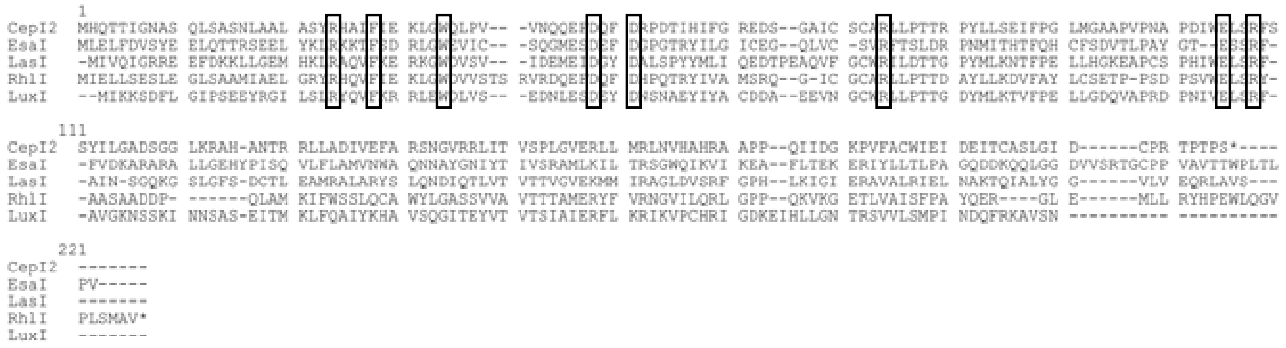

Supplementary Figure 1: Multiple-sequences alignment of CepI2 and selected AHL Synthases. EsaI (*Pantoea stewartii*), LasI and RhlI (*Pseudomonas aeruginosa* PAO1), LuxI (*Aliivibrio fischeri*) were used to align CepI2 (Bamb_6053) from *B. ambifaria* AMMD. Conserved residues are boxed in **black**, using EsaI as a reference (Arg24, Phe28, Trp34, Asp45, Asp48, Arg68, Glu97, Arg100).

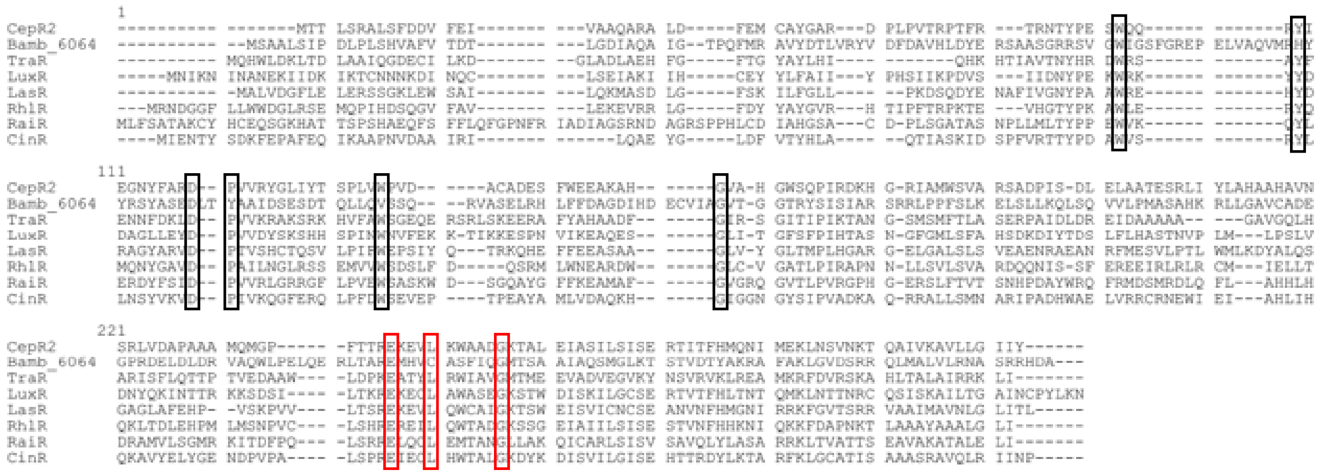

Supplementary Figure 2: Multiple-sequence alignment of CepR2 and selected LuxR. TraR (*Agrobacterium tumefaciens* C58), LuxR (*Aliivibrio fischeri*), LasR and RhlR (*Pseudomonas aeruginosa* PAO1), CinR (*Rhizobium etli*) were used to align CepR2 (Bamb_6040) and Bamb_6064 from *B. ambifaria* AMMD. Conserved residues are boxed, in **black** residues needed for AHL fixation (W57, Y61, D70, P71, W85, G113) and in **red** residues needed for DNA-binding (E178, L182, G188 with respect to TraR).

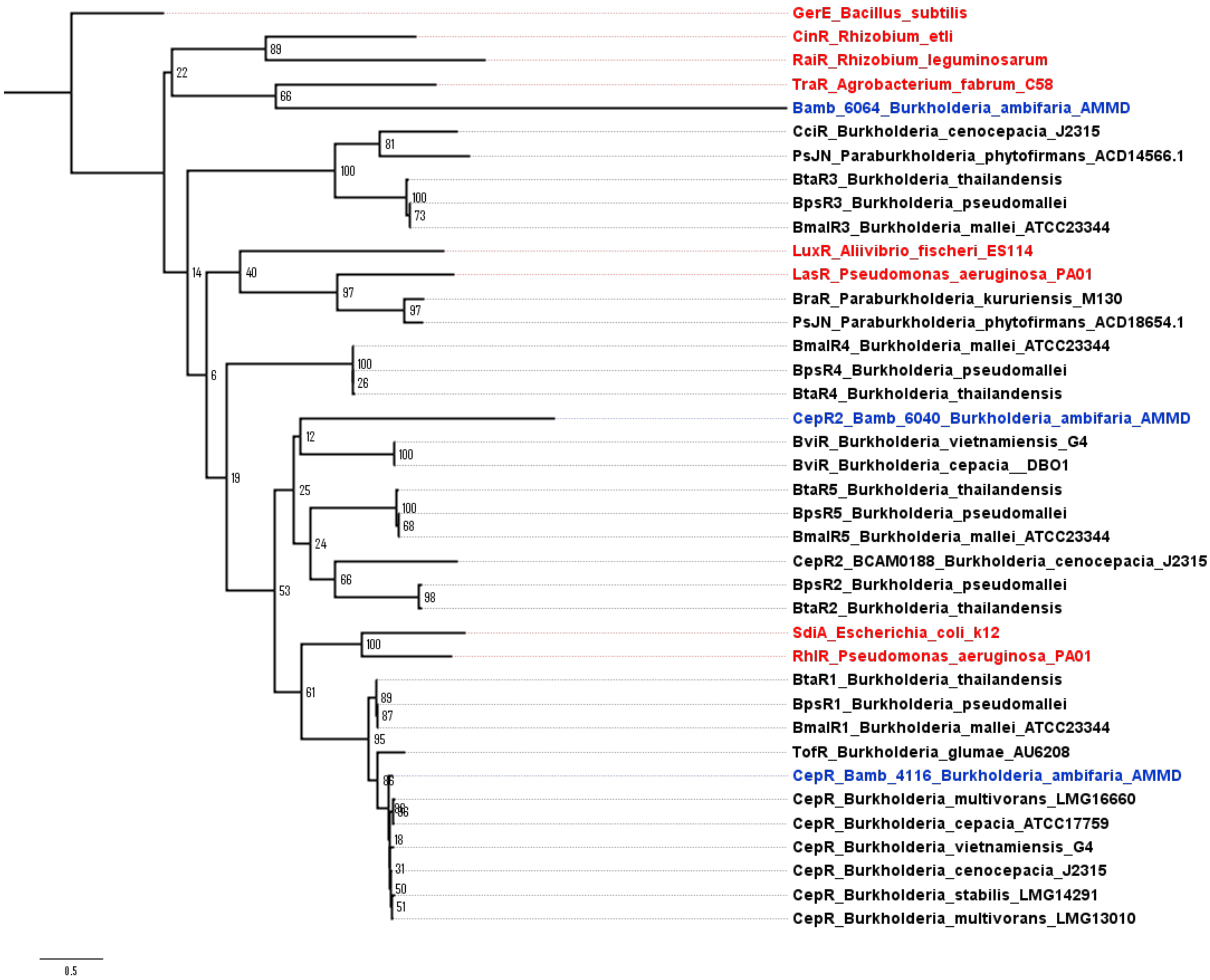

Supplementary Figure 3 : Phylogenetic tree of described *Burkholderia* LuxR

Names in **red** correspond to various LuxR, used as reference (CinR from *Rhizobium etli*, RaiR from *Rhizobium leguminosarum*, TraR from *Agrobacterium tumefaciens* C58, LuxR from *Aliivibrio fischeri*, LasR and RhlR from *Pseudomonas aeruginosa*, SdiA from *Escherichia coli* K12). In **black**, published and described LuxR of *Burkholderia* genus. In **blue**, *Burkholderia ambifaria* AMMD LuxR. The tree was inferred by using the Maximum Likelihood method and node labels correspond to boostrap values, and tree is rooted by GerE (*Bacillus subtilis*).

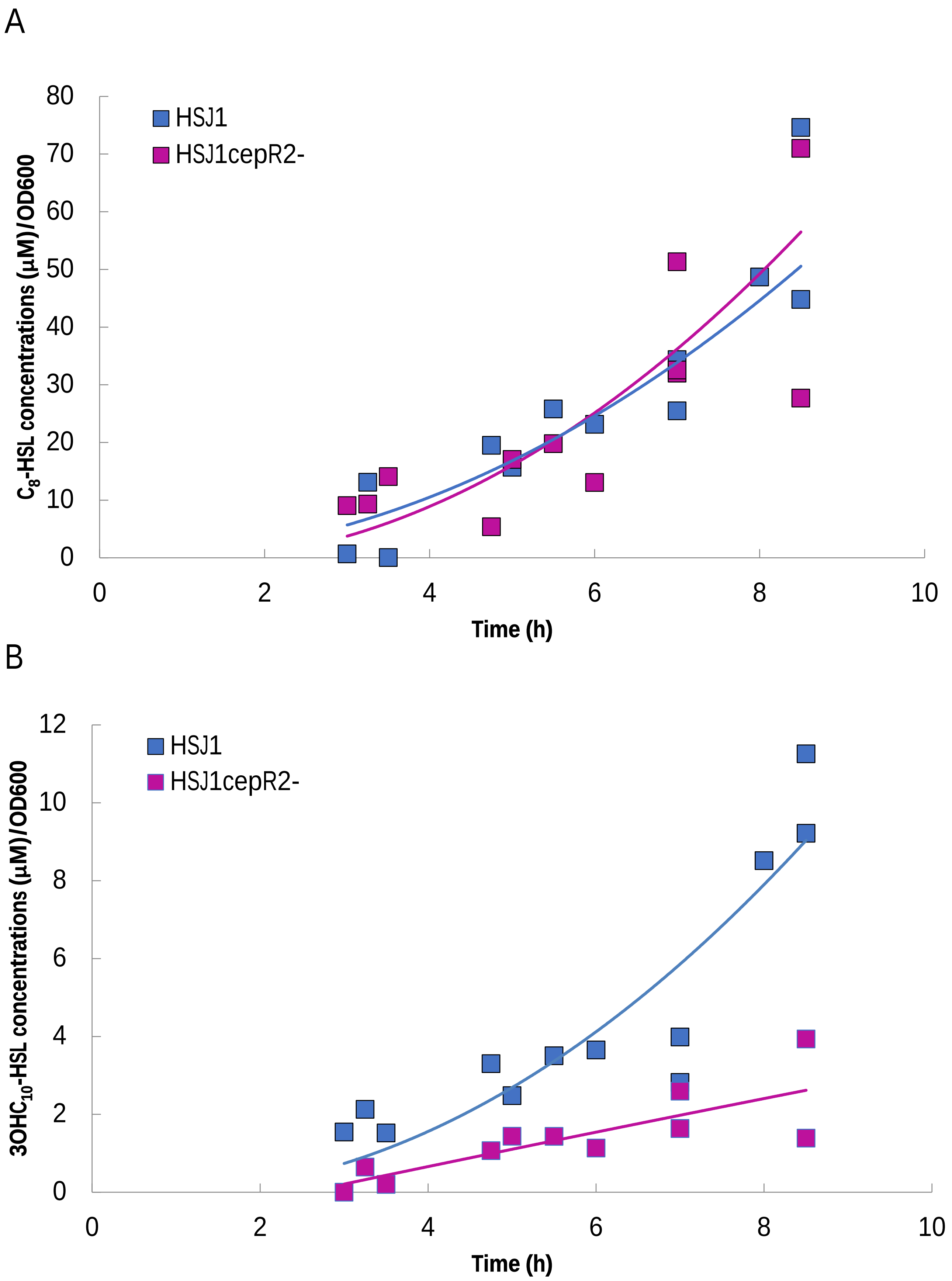

Supplementary Figure 4 : C_8-HSL (A) and $3OHC_{10}$-HSL (B) production in HSJ1 *cepR2*- and HSJ1 WT. Each point represents an extraction performed on one culture at a given time point.

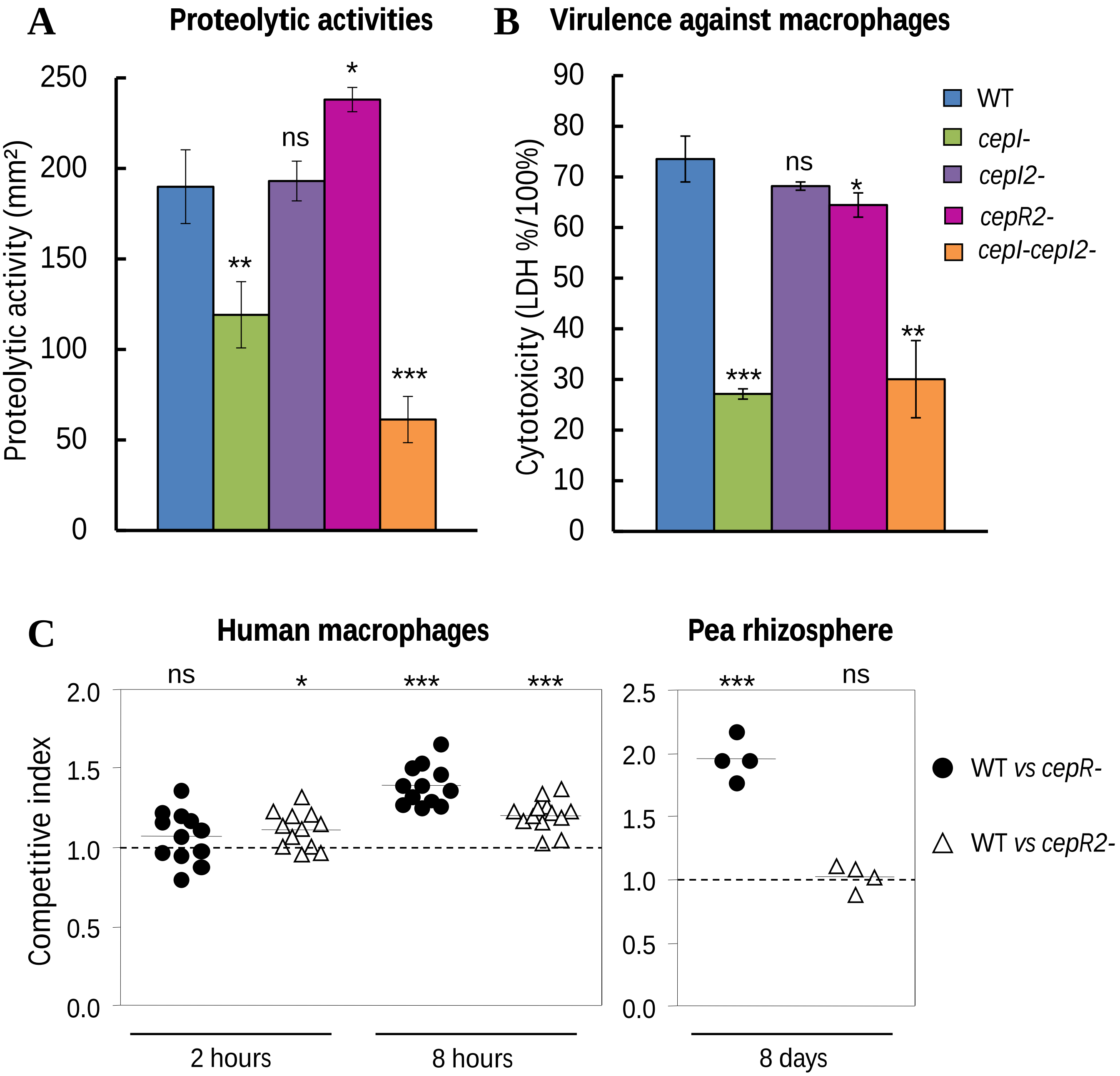

Supplementary Figure 5 : Phenotypic characterization of quorum sensing mutants in *B. ambifaria* HSJ1. (A) Proteolytic activity was estimated by measuring the degradation halo on milk agar plate after 24 h. **(B)** Virulence against human monocytes/macrophages THP1 cell line was estimated by measuring LDH activity in supernatants after 24h post infection at a MOI = 1. Results are expressed as percentage compared to a positive control (100% lysis). Data are expressed as means of at least three values +/- SD. **(C)** Competitive index (CI) analyses of *B. ambifaria* WT, *cepR*- and *cepR2*- mutants in human monocytes/macrophages THP1 cell line and in pea rhizosphere. CI for the entry in macrophages is defined as the ratio between the two strains in the input (intracellular bacteria after 2h treatment with antibiotics) divided by their ratio in the inoculum (t = 0h). CI for the proliferation in macrophages is defined as the ratio between the two strains in the output (intracellular bacteria after 8h treatment with antibiotics) divided by their ratio at the input (t = 2h). CI for colonization of the pea rhizosphere is defined as the CFU output (day 8 after inoculation) ratio of the two strains divided by the CFU input (inoculum) ratio. The wild-type is more competitive than *cepR*- or *cepR2*- when the CI is greater than 1. The bars represent the median value for each group. ***, $p < 0.001$; **, $p < 0.01$; *, $p < 0.05$; ns, non-significant.

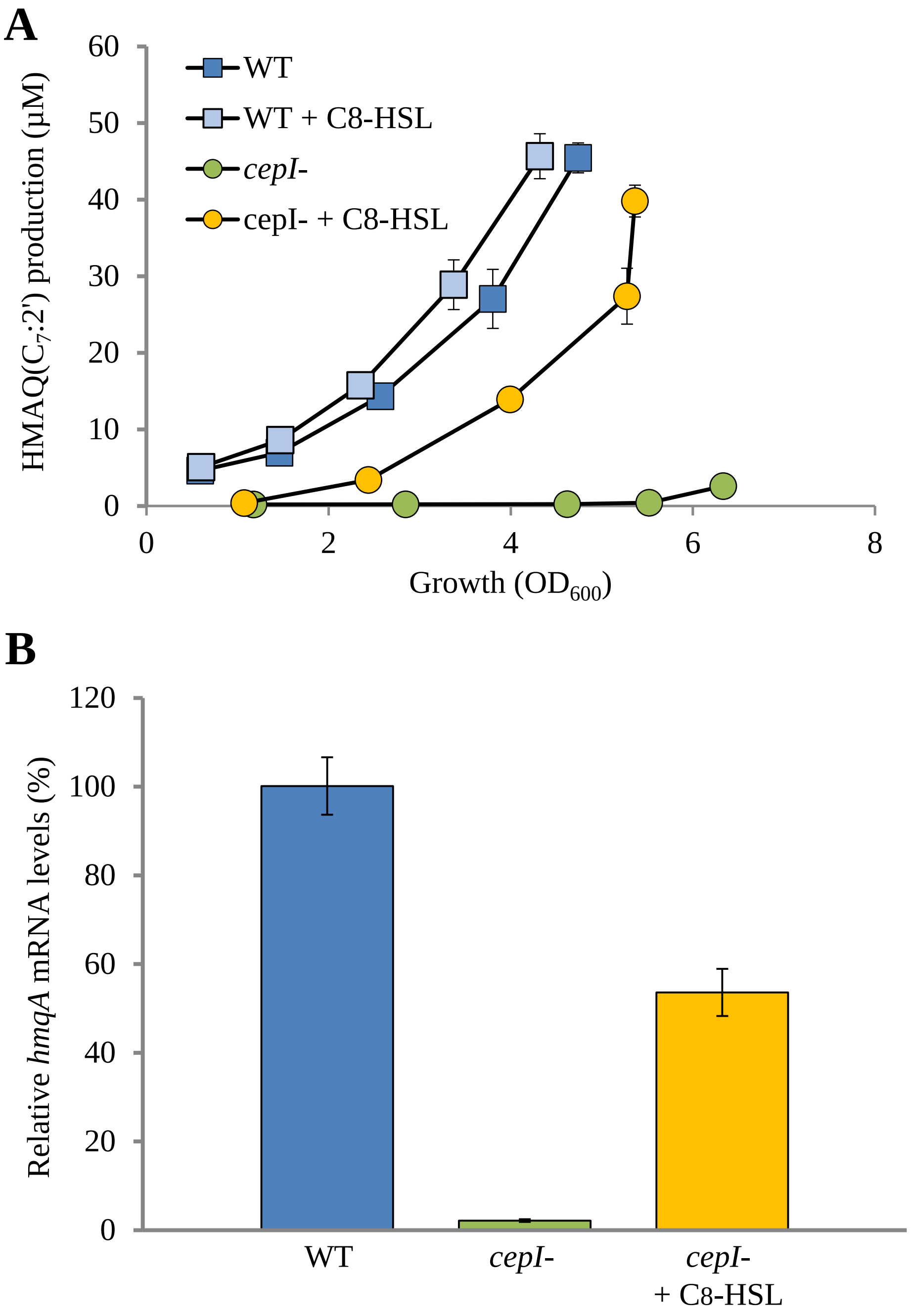

Supplementary Figure 6: Impact of C_8-HSL supplementation on HMAQ production and on *hmqA* gene expression in *B. ambifaria* HSJ1 WT and *cepI*- mutant strains. WT and *cepI*- were analyzed for **(A)** HMAQ-C_7:2’ production using LC/MS, in presence or absence of exogenously added C_8-HSL. Values are presented as relative concentration compared to the internal standard HHQ-d4. Results are expressed as means ± SD of three replicates. **(B)** Relative expression of the *hmqA* gene was determined by quantitative reverse transcription PCR (qRT-PCR) in HSJ1 WT, *cepI*- mutant and *cepI*- mutant supplemented with C_8-HSL. The results are presented as relative quantification of gene expression compared to the WT normalized to 100%. The results are expressed as means ± SD of three replicates.

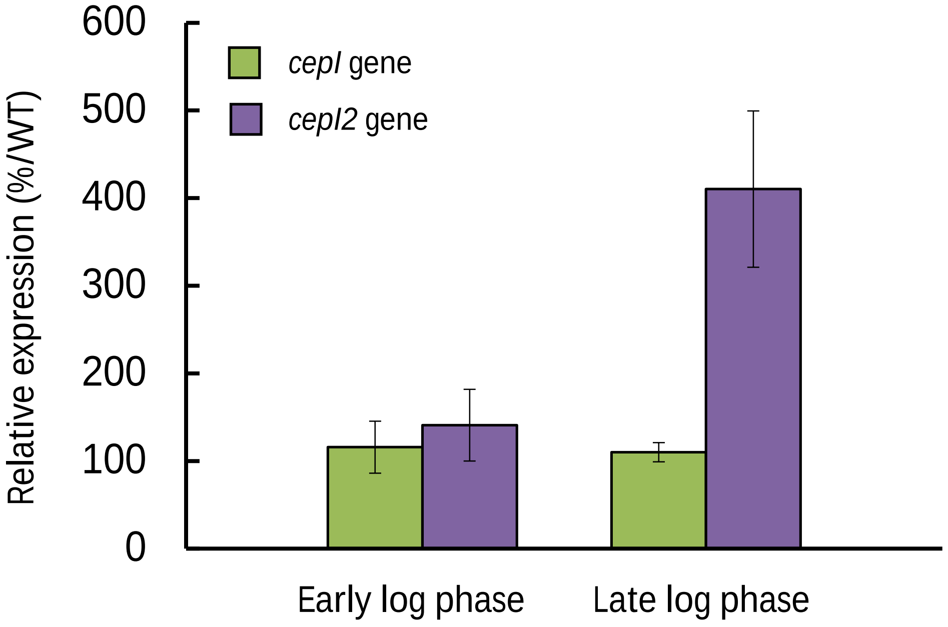

Supplementary Figure 7: Expression of *cepI* and *cepI2* genes in the *hmqA*- mutant at the early and late log phase.

Relative expression of *cepI* and *cepI2* genes in *hmqA*- mutant compared to the WT at the beginning and the end of log phase in TSB liquid cultures. Values are expressed as means ± SD of three replicates.
